# The politics of glyphosate regulation: lessons from Sri Lanka’s short-lived ban

**DOI:** 10.1186/s12992-023-00981-2

**Published:** 2023-11-13

**Authors:** Tim Dorlach, Sandya Gunasekara

**Affiliations:** 1 University of Bayreuth, Fritz-Hornschuch-Str. 13, Kulmbach, 95326 Germany; 2 The University of Queensland, Brisbane, QLD 4072 Australia

**Keywords:** CKDu, Environmental health, Global South, Glyphosate, Multiple streams framework, Pesticides, Policy reversal, Public health, Sri Lanka

## Abstract

**Background:**

Glyphosate is the world’s most used herbicide and a central component of modern industrial agriculture. It has also been linked to a variety of negative health and environmental effects. For instance, the International Agency for Research on Cancer classified glyphosate as “probably carcinogenic to humans” in 2015. This has motivated widespread political demands for stricter glyphosate regulation but so far few governments have followed through.

**Methods:**

We conduct a case study of Sri Lanka, which in 2015 became the first and so far only country in the world to adopt and implement a complete glyphosate ban. But this ban proved to be short-lived, as it was partially reversed in 2018 (and later fully revoked in 2022). To explain the political causes of Sri Lanka’s pioneering glyphosate ban and its subsequent reversal, we employ process tracing methods drawing on publicly available documents. Our analysis is theoretically guided by the multiple streams framework and the concept of self-undermining policy feedback.

**Results:**

Glyphosate regulation rose to the top of the Sri Lankan political agenda in 2014 when a local scientist linked glyphosate exposure to an epidemic of Chronic Kidney Disease of Unknown Origin (CKDu). A glyphosate ban was eventually adopted in June 2015 by the newly elected government of Maithripala Sirisena. The ban was a political commitment made to the Buddhist monk Rathana Thero and his party, which had supported Sirisena during his presidential campaign. The ban’s partial reversal in 2018, implemented through sectoral exceptions, was the result of continued lobbying by export-oriented plantation industries and increased political concerns about potential negative effects on the large and structurally powerful tea sector. The reversal was further aided by the scientific community’s failure to corroborate the hypothesized link between glyphosate and CKDu.

**Conclusions:**

The case of Sri Lanka suggests that strict glyphosate regulation becomes more likely when coupled with locally salient health risks and when decision-making authority is de-delegated from regulatory agencies back to the political executive. Meanwhile, the short-lived nature of the Sri Lankan ban suggests that strict glyphosate regulation faces political sustainability threats, as the apparent lack of cost-effective alternative herbicides motivates persistent business lobbying for regulatory reversal.

**Supplementary Information:**

The online version contains supplementary material available at 10.1186/s12992-023-00981-2.

## Introduction

Glyphosate has become the world’s most common herbicide over the past two to three decades.^1^ Herbicides are a group of pesticides that target unwanted plants (or weeds) and are an essential component of modern industrial agriculture [11]. Glyphosate was first patented as a herbicide in the early 1970s and has since been marketed under the trade name Roundup by the American transnational agribusiness corporation Monsanto [22]—which was acquired by Germany’s Bayer in 2018. Global agricultural glyphosate use has grown rapidly since the 1990s, driven by the introduction of several genetically modified, glyphosate-tolerant crops and the expiration of Monsanto’s glyphosate patent in 2000 ([11], pp. 4–6). Proponents have described glyphosate as an almost “perfect”, “once-in-a-century” herbicide, given its broad-spectrum effectiveness and synergies with genetically modified crops, while supposedly being “toxicologically and environmentally safe” ([22], p. 319). Indeed, there is little doubt that glyphosate use massively boosts agricultural productivity, at least on the short term, and it is generally considered as less harmful than other herbicides such as paraquat. As a result, glyphosate use has long remained broadly accepted and loosely regulated.

Over the past decade, however, concerns have grown about potentially wide-ranging side effects of glyphosate use on human health and the environment [45, 51, 70]. A watershed moment came in March 2015, when the International Agency for Research on Cancer (IARC) classified glyphosate as “probably carcinogenic to humans” ([29], p. 491), with particular reference to a type of cancer known as non-Hodgkin’s lymphoma. While other agencies, most notably the European Food Safety Authority (EFSA) and the Environmental Protection Agency (EPA) of the United States, came to different conclusions [3, 5], IARC’s classification suddenly increased the global salience of the glyphosate issue and motivated a variety of political responses. In the European Union (EU), IARC’s decision motivated several member states to restrict glyphosate use domestically and oppose a long-term renewal of glyphosate’s license in the EU, currently set to expire in December 2023 [3, 68]. In the United States, the IARC decision triggered a series of lawsuits by “people who believed they had been injured by the herbicide” ([42], p. 319), a large portion of which Monsanto/Bayer settled for 10 billion USD in 2020 (Source 1, abbrev. S1).^2^

While there has been rapid growth in research on the politics of glyphosate in the Global North, especially since the 2015 IARC classification [2, 3, 14, 30, 40, 66–68, 71], research on glyphosate regulation in the Global South remains much more limited (but see [1]). This is especially puzzling given that Southern countries, including Argentina, Colombia, El Salvador, and Sri Lanka, were among the first to seriously consider restricting glyphosate use, and did so before the 2015 IARC decision.

To contribute to a better understanding of the politics of glyphosate regulation, this article studies the case of Sri Lanka, a democratic lower-middle income country of 22 million people in South Asia. In 2015, Sri Lanka became the first country in the world to completely ban glyphosate, attracting significant global attention. However, the ban was short-lived, as it was partially reversed in 2018, when an exception for the tea and rubber industries was introduced, and eventually revoked in 2022 in the context of a broader economic and political crisis (see [16, 53]). The Sri Lankan case therefore provides us with a unique opportunity to examine the political causes and processes that can bring about stricter glyphosate regulation but also those that can prevent it. This article provides the first comprehensive scholarly analysis of the politics behind Sri Lanka’s short-lived glyphosate ban (but see [74]).

Based on an analysis of publicly available documents, this article traces the political processes behind Sri Lanka’s 2015 glyphosate ban and its 2018 partial reversal. In a nutshell, we find that a glyphosate ban forced itself on the Sri Lankan political agenda after studies had linked growing use of glyphosate to the prevalence of Chronic Kidney Disease of Unknown Origin (CKDu), a poorly understood chronic illness that has burdened rural farming communities and the country’s healthcare system since the 1990s. Public pressure translated into swift, large-scale policy change as it mounted during a competitive presidential campaign, in which the issue of glyphosate regulation gained particular importance with the electoral constituency and key political allies of the eventual winner of the election, Maithripala Sirisena, whose government passed the national glyphosate ban soon after it took office. At the time, the forces in favor of a glyphosate ban were sufficient to countervail the powerful interests of Sri Lanka’s export-oriented plantation sector. The partial reversal of the glyphosate ban in 2018, through the introduction of exceptions for the tea and rubber sectors, came after a crushing election defeat and was the result of continued lobbying by export-oriented plantation industries and increased political concerns about potential negative effects on the large and structurally powerful tea sector. The reversal was further aided by the failure of the scientific community to corroborate the hypothesized link between glyphosate and CKDu.

### Background: the global politics of glyphosate regulation

Through the first four decades after the herbicide’s initial market introduction in 1974 in the United States ([22], p. 319), glyphosate regulation remained an issue of “quiet politics” [13], that is, of low political salience, and thus dominated by interest groups and regulatory agencies. It should be noted that, in the 1980s, Monsanto and the EPA debated the potential carcinogenicity of glyphosate on the basis of an ostensibly ambiguous mouse study, but Monsanto eventually managed to convince the EPA to drop its demand for a repeat study ([25], pp. 31–39; [56], p. 535). Despite this early regulatory controversy, glyphosate remained loosely regulated in the United States and the rest of the world.

Global glyphosate use has grown immensely in recent decades. Between 1995 and 2014, global use of glyphosate grew almost 15-fold, from 51 to 747 million kilogram ([4], p. 6). From a political economy perspective, two factors are key to understanding “why glyphosate-based agricultural herbicides have become so entrenched in modern agriculture” ([11], p. 1). First, during the 1980s and 1990s glyphosate was “repurposed” from a herbicide that killed plants relatively indiscriminately to a product that could be used as part of a so-called “technological package” together with glyphosate-tolerant genetically modified crops, such as Monsanto’s glyphosate-tolerant (“Roundup Ready”) soy, maize, canola and cotton. Second, after the expiration of Monsanto’s glyphosate patent in 2000, there has been a massive growth of generic glyphosate production, especially in China, offering lower-cost alternatives to Roundup ([11], pp. 4–6; also see [64, 72]). These developments have contributed to the intense glyphosate reliance of global industrial agriculture that we can witness today.

Since the 2010s, the global political salience of glyphosate regulation has soared, making it an issue increasingly shaped by public opinion and political parties. A first key event in this process was the so-called “Séralini affair”. In 2012, the French biologist Gilles-Éric Séralini, who had long been interested in the potential negative effects of genetically modified crops and pesticides, published a controversial paper in the peer-reviewed journal *Food and Chemical Toxicology*. It reported on an experiment that tested the health effects on rats of a diet containing genetically modified maize and glyphosate (both produced by Monsanto). The paper found increased mortality among rats that consumed genetically modified maize, glyphosate, or both, mostly due to tumor formation and kidney insufficiencies. The publication of the paper was accompanied by a “tightly orchestrated media offensive”, including a complementary book and documentary film ([9], p. 158). Immediately after publication, the methods and findings of the paper came under intense criticism, most notably from Monsanto and various scientists instigated by Monsanto ([26], p. 5). The journal eventually retracted the paper in November 2013, following active lobbying from Monsanto and under a clear conflict of interest of its editor, who had recently accepted a consulting job with Monsanto (see [26], pp. 5–6; [42], pp. 321–322).^3^ Despite its retraction and regardless apparent methodological deficiencies, the Séralini paper contributed to increasing the political salience of glyphosate (and GMO) regulation (S2).

Another important global event that increased the political salience of glyphosate regulation occurred in March 2015, when the International Agency for Research on Cancer (IARC), the WHO’s specialized agency for the evaluation of cancer hazards [58], classified glyphosate as “probably carcinogenic to humans” ([29], p. 491; see [12]), making special reference to a type of cancer known as non-Hodgkin’s lymphoma. IARC’s assessment and decision was based on a review of openly available, mostly published studies on the topic, highlighting the significance of the retraction and subsequent republication of the Séralini paper (S3, p. 12).^4^ Monsanto responded to IARC’s decision with a well-documented public affairs campaign to undermine the agency’s reputation for independence (S4, see [48]).

While without formal consequences, IARC’s 2015 decision put significant pressure on regulators around the world to justify or reconsider their lenient approach to glyphosate regulation. In October 2015, EFSA affirmed that “glyphosate is unlikely to pose a carcinogenic hazard to humans” [67], an assessment subsequently also confirmed by the European Chemicals Agency (ECHA). Likewise, the EPA concluded in September 2016 that glyphosate was “not likely to be carcinogenic to humans” [5]. The discrepancy in the risk assessments of IARC on one side and regulatory agencies such as EFSA and EPA on the other has since puzzled many observers. The disagreement can in part be attributed to the different methodologies with which these agencies select and evaluate relevant evidence. For instance, IARC generally limits its reviews to studies that have been published or accepted for publication, emphasizing transparency, while EFSA also considers confidential industry studies, prioritizing completeness ([7], p. 202). That being said, there have also been concerns about undue corporate influence, in particular by Monsanto, on EFSA’s risk assessment of glyphosate (S5-6).

Despite the insistence of major regulatory agencies that glyphosate was safe, public opposition began to grow, especially in Europe. In 2017, the European Citizens Initiative “Ban glyphosate and protect people and the environment from toxic pesticides” collected more than one million signatures ([68], p. 608). Several member states started opposing an EU-level renewal of glyphosate’s authorization, leading to a deadlock in the EU’s relevant committees [67]. Glyphosate’s authorization in the EU is currently set to expire in December 2023 and it remains unclear how the stalemate will be resolved (see S7). Meanwhile, several EU member states, including Austria, France and Germany, have announced plans to comprehensively restrict glyphosate use at the national level ([66], see S8). However, none of these countries has yet officially adopted, let alone implemented, a full ban on glyphosate including agricultural uses.

In the Global South, glyphosate use and its potential health and environmental risks have also become increasingly politicized, in some instances since well before IARC’s 2015 decision. In Argentina and Brazil, for instance, the issue of glyphosate is salient because both countries are global leaders in GMO cultivation, which is closely linked to intensive agricultural glyphosate use [49]. In Argentina, mobilization by affected communities that began in the early 2000s led to significant local restrictions on glyphosate use, including the controversial technique of aerial spraying ([1], p. 289). At the national level, however, glyphosate regulation remains lenient in both Argentina and Brazil [54]. In a different context, the Colombian government had used extensive aerial spraying of glyphosate since the early 1990s to prevent coca cultivation and drug trafficking. While the practice had long been politically contested, including by severely affected indigenous communities, it was halted only after IARC’s landmark decision of 2015 (S9). More recently, and apparently motivated at least in part by the IARC decision, Thailand planned to fully ban glyphosate as of December 2019, but soon reversed its decision after interventions by Monsanto and the United States government as well as reports of protests by local farmer groups (S10-12).

## Methods

To contribute to a better understanding of the politics of glyphosate regulation, we conduct a case study [75] of Sri Lanka’s 2015 glyphosate ban and its subsequent reversal. In June 2015, Sri Lanka became the first country in the world to completely ban glyphosate, significantly before similar policy changes occurred in any other country. From a methodological perspective, this makes Sri Lanka an “index case” of strict glyphosate regulation—i.e., the “first instance” of this particular “phenomenon” ([23], p. 398)— and thus a privileged setting to explore its causes. Given that glyphosate had not been banned by other countries at the time, the Sri Lankan case allows us to largely discount case interdependence and thus “horizontal” policy diffusion as an explanation, and instead focus on its endogenous causes or potential vertical policy diffusion, e.g., from international organizations such as IARC (see [23], pp. 398–399; [65]). The partial reversal of Sri Lanka’s glyphosate ban in May 2018 provides us an opportunity to also examine the political causes of the loosening of glyphosate regulation.

To explain Sri Lanka’s 2015 glyphosate ban and its subsequent reversal, we use inductive process tracing, a method that is well-suited for studying the causes of specific policy outcomes as well as theory building ([69], pp. 443–445; see [38]). While our approach to process tracing is more inductive, it is still guided by relevant existing theories (outlined in the next paragraph). As argued by Trampusch and Palier ([69], p. 445), “inductive analysis of processes does not merely consist of naïve observations of empirical events from which theoretical ideas are derived, but rather forms a theoretically informed analysis (= decomposition) of processes that looks for causal chains between the observed events”. We complement our process tracing analysis with two within-case comparisons (see [44], p. 53), comparing Sri Lanka’s glyphosate ban introduced in 2015 with an earlier, failed policymaking attempt in 2014 and with the ban’s subsequent partial reversal in 2018.

Our case study is theoretically guided by the multiple streams framework of the policy process [39, 76], which we amend to also account for policy reversal. We first use a standard version of the multiple streams framework to guide our explanation of Sri Lanka’s pioneering glyphosate ban, as the framework is considered ideal for explaining “how policies are made by national governments under conditions of ambiguity” ([76], p. 65). The framework assumes that national policy systems are characterized by distinct *problem*, *policy*, and *politics streams*. Policy change becomes much more likely when these three separate streams are *coupled*, which usually happens during *policy windows* and through the agency of *policy entrepreneurs* ([76], p. 65). While this framework is regularly used for the analysis of policy formation (i.e., agenda setting and policy adoption), it has, to the best of our knowledge, not yet been used to also examine subsequent policy reversals. We therefore make two amendments to the standard framework. First, we draw on the idea of *decoupling*, especially of “problems from solutions”, previously developed for the analysis of implementation failure ([77], p. 64), which we apply to the phenomenon of policy reversal. Second, we also incorporate the concept of *self-undermining policy feedback*, which describes a set of “endogenous forces of policy revision”, including the emergence of “unanticipated policy losses for powerful groups” ([33], pp. 442, 444). Together, the concepts of decoupling and self-undermining policy feedback help guide our explanation of why Sri Lanka’s pioneering glyphosate ban proved to be short-lived.

Our analysis draws on data collected during remote field research [31] conducted in both English and Sinhalese between 2022 and 2023. We collected publicly available documents from a broad range of sources. In particular, we conducted focused, iterative internet searches of the archives of major Sri Lankan news websites (e.g., Ada Derana, Colombo Page, Colombo Telegraph, Daily FT, Daily Mirror, Daily News, EconomyNext, Sunday Observer, Sunday Times) and government websites (e.g., gazette.lk, news.lk). To make our data sources more transparent [50], we present complete references to all our primary sources in the Additional file 1. To minimize the problem of “reference rot”, all links to primary online sources have been archived with Perma [24].

## Results

### Political, economic and regulatory context

Sri Lanka is a South Asian island nation with a population of 21 million in 2015. It used to be a British colony, known as Ceylon, from the early nineteenth century until 1948, when it gained independence. Since then, Sri Lanka has been a representative democratic republic with a semi-presidential, multi-party system. Modern Sri Lankan politics have been profoundly shaped by a civil war, lasting from 1983 to 2009, between Sri Lanka’s Tamil minority and its Sinhalese dominated government and the associated development of Sinhalese Buddhist nationalism as the country’s dominant political ideology, including the emergence of political Buddhism, that is, the active engagement of Buddhist monks in politics, most notably through the 2004 founding of the National Heritage Party (*Jathika Hela Urumaya*, JHU) [15, 19]. While the monks’ nationalist, anti-Western ideology and their advocacy for a military solution to the government’s conflict with the Tamils is well-established [19], it is relevant for our analysis that at least some of them also have strong environmentalist policy preferences (see [15], p. 88). Since the mid-2000s, Sri Lankan politics have been especially dominated by the Rajapaksa family [17]. Mahinda Rajapaksa was president from 2005 to 2015, gaining fame and broad public support for (violently) ending the country’s civil war in 2009, and prime minister from 2019 to 2022, under the presidency of his brother Gotabaya Rajapaksa. Notably, the glyphosate ban that this article focuses on was introduced during the brief non-Rajapaksa interregnum from 2015 to 2019.

Sri Lanka has embraced a market- and export-oriented economic model since the late 1970s. Today, it is a middle-income country at the threshold of upper-middle income status. After a decade of strong growth, it reached a GDP per capita of 4060 USD in 2015. The Sri Lankan economy is currently dominated by international tourism, export-oriented textile and plantation sectors, as well as rice production for the local market. Tea has long been Sri Lanka’s single most important export product, worth 1.3 billion USD or 12% of all exports in 2015 (S13, p. 7). At the same time, the tea sector has also been the largest recipient of glyphosate imports, followed by the corn and rice sectors ([47], p. 249). Alongside conventional agriculture, Sri Lanka has developed a small but growing sector of organic agriculture, “catering mostly to growing niche markets in the West”, including “tea and coconut products, rice, fruits, spices and extracts” (S14). Notably, Sri Lanka allows neither local production nor import of genetically modified (GM) organisms (S15).

The sale and use of pesticides in Sri Lanka is governed by the 1980 Control of Pesticides Act and subsequent regulations. This legislation established a system of “delegated authority”, in which the Pesticides Technical Advisory Committee provides “technical advice and decisions regarding the registration and regulation of pesticides”, while the Registrar of Pesticides, a position in the Department of Agriculture, administers procedures and implements decisions ([59], pp. 65, 58). In the 2000s, on the eve of the glyphosate ban that we are focusing on, pesticide policymaking in Sri Lanka was dominated by a “tightly integrated” “epistemic community” that had “links with industry” but was largely “free from political interference” ([59], pp. 62, 64). One major policy change in this period was the 2008 decision to phase out the use of the herbicide paraquat (banned in the European Union since 2007), due to strong links with a particular public health crisis [59]: in the 1990s, Sri Lanka had one of the highest suicide rates in the world, especially in rural communities, where most suicides were through self-poisoning with highly toxic pesticides, most notably paraquat [28, 59]. While the paraquat ban did help to reduce suicide rates [41], it also further increased farmers’ reliance on other herbicides, especially glyphosate [47].

### Sri Lanka’s 2015 glyphosate ban

Despite earlier debates about the risks of pesticides, and glyphosate in particular, the introduction of Sri Lanka’s 2015 glyphosate ban needs to be understood against the backdrop of a “mysterious” kidney disease epidemic [10, 60, 62], which was the main *problem* the glyphosate ban addressed. Chronic Kidney Disease of Unknown Origin (CKDu) is, as its name suggests, a type of kidney disease that remains poorly understood and cannot be explained by the causes of common chronic kidney disease (such as diabetes and high blood pressure). CKDu disproportionally affects agricultural communities, especially across Central America and South Asia. In Sri Lanka, CKDu emerged in the early 1990s and became especially prevalent among rice farming communities in the districts of Anuradhapura and Polonnaruwa in the country’s North Central Province. In 2010, Sri Lanka’s health ministry launched a research project to investigate, in collaboration with the WHO, the causes of CKDu. The project team later concluded that chronic exposure to cadmium “through the food chain”—via vegetables from phosphate fertilizer and via freshwater fish—might be “a causative factor for CKDu in Sri Lanka” ([34], pp. 1, 10).

The issue of glyphosate regulation rapidly entered the Sri Lankan political agenda when it became coupled with the problem of the CKDu epidemic by a local policy entrepreneur. In February 2014, Channa Jayasumana, then a medical doctor and researcher at Rajarata University (located in the CKDu-affected North Central Province),^5^ published a study that associated the prevalence of CKDu with glyphosate use [36]. More specifically, Jayasumana argued that glyphosate was “Compound-X” ([36], p. 2138) in that it “bonds with toxic heavy metals in the environment such as cadmium and arsenic, forming stable compounds that are consumed in food and water and do not break down until reaching victims’ kidneys” (S16). Jayasumana had previously conducted his dissertation research and published several articles about the potential causes of CKDu, but this 2014 paper was the first to explicitly link CKDu with glyphosate—albeit admittedly as a “hypothesis” with “limited testing” ([36], p. 2140; but see [37]).

It should be noted that Jayasumana’s glyphosate hypothesis emerged from a distinct political ideology and scientific community. Jayasumana has long been closely associated with Sinhala-Buddhist nationalism and the specific ideology of *jathika chinthanaya* (national consciousness) ([61], p. 13; see [20], esp. p. 51, fn. 14). Both his scientific and later political work has been characterized by an “indigenous discourse […] shaped by a sense of colonial injury and fashioned as an anti-western response to the denigration of ‘local’ knowledge” ([61], p. 3). Jayasumana’s research on CKDu emerged under the mentorship of Nalin de Silva (see [35], p. 72), a controversial academic who had previously linked CKDu with arsenic in water, an insight he claimed to have received from “God Natha”, a local guardian deity ([61], p. 13). Jayasumana’s 2014 paper was co-authored with a member of Hela Suwaya, a Buddhist civil society organization promoting indigenous agriculture and medicine, which is also associated with worship of “God Natha” (S17-18). These ideological associations also had methodological implications, as Jayasumana’s 2014 paper explicitly acknowledged that “according to the prevailing Buddhist philosophical values within the country, no animal models were used in the current study” ([35], p. 2140). None of this, to be clear, disproves Jayasumana’s glyphosate hypothesis, but it shows that it emerged in a distinct, non-Western scientific community, which might even help explain its subsequent political influence.

Jayasumana’s glyphosate hypothesis gained immediate public attention in Sri Lanka.^6^ Industry reacted promptly, reportedly publishing “a full-page paper advertisement in all national newspapers demanding withdrawal of [Jayasumana’s] findings within two weeks if not they will go the court” (S19, p. 9). In March 2014, and with direct reference to the Jayasumana paper published just a few weeks earlier, Mahinda Rajapaksa, Sri Lanka’s President at the time, “ordered the immediate removal of Glyphosate from the local market” (S22). The ban was soon (in April 2014) placed on hold, however, after opposition from the private sector and the governmental Office of the Registrar of Pesticides, who noted that “the ban rests on a theory that has not been proven” (S16).

A *policy window* for comprehensive glyphosate regulation emerged in November 2014, when President Mahinda Rajapaksa announced early presidential elections for January 2015, seeking an unprecedented third term in the context of worries over increasing authoritarianism.^7^ This led Maithripala Sirisena, Rajapaksa’s health minister, to resign from his posts in Rajapaksa’s government and announce that he would challenge Mahinda Rajapaksa in the elections, quickly becoming the common opposition candidate. Among the groups that defected from Rajapaksa’s coalition to support Sirisena was the Jathika Hela Uramaya (JHU) party and its parliamentary leader Athuraliye Rathana Thero (S24), who later cited Rajapaksa’s failure to implement the glyphosate ban as a major reason for this shift (S25). It is also relevant to note that Sirisena grew up in the CKDu-affected North Central Province and had long represented the Polonnaruwa district in parliament. During his campaign, Sirisena set up a relief fund for CKDu patients (S26), and his election manifesto promised to “immediately prohibit the import and distribution of agro-chemicals that were identified as causing kidney diseases” (S27). Despite these pronouncements, Sirisena’s presidential bid was clearly dominated by his promise to end the ten-year rule of Mahinda Rajapaksa and his family and to reform Sri Lanka’s “authoritarian executive presidential system” (S28).

The policy window for a glyphosate ban firmly opened when Sirisena surprisingly won the presidential election of January 2015 with 51% of the vote. The first months of the Sirisena government were especially reform-oriented, given that Sirisena had promised early parliamentary elections (to renew and strengthen his mandate), which were eventually called in June and held in August 2015 (S29). Addressing the problem of CKDu clearly was one of Sirisena’s priorities. In February 2015, Sirisena launched the extrabudgetary National Kidney Trust Fund that would help finance a monthly cash transfer (allowance) program targeting kidney patients as well as CKDu-related infrastructure investments (S30-33). In March 2015, during a state visit to China, Sirisena successfully requested substantial Chinese development aid for building a large specialized kidney hospital (S34-35), which was subsequently built and recently opened in Polonnaruwa (S16), a center of the CKDu epidemic but also Sirisena’s hometown and electoral district. Sirisena also relaunched the presidential task force on kidney disease prevention (S37-38).

At the same time, pressure on glyphosate continued to grow. The Sri Lankan researcher Channa Jayasumana published another paper in January 2015 that appeared to empirically corroborate his glyphosate hypothesis of CKDu [37]. Meanwhile, IARC’s March 2015 classification of glyphosate as “probably carcinogenic to humans” provided an additional rationale for glyphosate regulation, even though it did not weigh in on the potential link between glyphosate and CKDu (given that CKDu is not a cancer).

Eventually, in May 2015, and with direct reference to Jayasumana’s research, President Sirisena announced that he would “totally ban the import and usage of Glyphosate pesticide” (S39), a decision that was implemented through a ministerial import ban in June 2015 (S40-41). By implementing the ban through an import rather than sale and use ban, the government bypassed Sri Lanka’s established system of delegated, technocratic pesticide regulation (see [59]). This worked because there is no domestic production of glyphosate-based herbicides in Sri Lanka. Foreshadowing subsequent debates over the needs of Sri Lanka’s export-oriented plantation sector, the government acknowledged that “a strong demand has been made that glyphosate was necessary for tea cultivation” but the cabinet still decided “to completely halt the import of Glyphosate” (S41).

The central question that emerges here is why the Sirisena government went through with a glyphosate ban while the previous Rajapaksa government had backpedaled a year earlier. After all, both faced largely the same evidence on the link between glyphosate and CKDu, while IARC’s 2015 decision did not appear to be a significant factor in the Sri Lankan political debate. The available evidence suggests that the Sirisena government’s decision to ban glyphosate was primarily a strategic concession to Rathana Thero and his JHU, who (as environmentalists) had a strong interest in the glyphosate issue and who were perceived as crucial allies by the Sirisena government. As noted above, political Buddhism in Sri Lanka has long-standing connections with conservationism and environmentalism, and Rathana Thero in particular has long been a vocal advocate of organic agriculture. Sirisena reportedly even offered Rathana Thero the post of environment minister in his government, which the latter turned down (S42). Rathana Thero would later cite Rajapaksa’s failure to implement the planned glyphosate ban as a major reason for his party’s 2015 shift in political allegiance from Rajapaksa to Sirisena (S25, S43). The Rajapaksa government had apparently felt comfortably in power in 2014, arguably significantly reducing pressure to make policy concessions to its then-ally JHU. In contrast, the JHU’s stance on glyphosate (and other policy issues) carried much more weight in the Sirisena government, as its alliance with the JHU had played a key role in splitting the Sinhala-Buddhist nationalist vote (S44) and was likely decisive in Sirisena’s slim election victory. In Multiple Streams Framework terms, Rathana Thero and his JHU therefore were the policy entrepreneurs that coupled the policy and the political streams.

An additional issue that needs to be considered to fully explain Sri Lanka’s adoption of a glyphosate ban is that the power of organized business interests was evidently insufficient to prevent the ban. Sri Lanka’s plantation industry had clearly opposed the glyphosate ban and had advocated, at the very least, for an exception for the tea sector. Sri Lanka’s broader agricultural sector, with a focus on rice production, was less organized and vocal, but also was not in favor of the ban. On the one hand, this clearly seems to confirm the theory that business power is lower in times of high issue salience (or “loud politics”) [13]. On the other hand, it also speaks to the somewhat lower (structural) power of pro-glyphosate advocates in Sri Lanka due to the country’s lower dependence on GM crops (see [52]). As discussed above, growth in global glyphosate use has been driven by the emergence of a “technological package” of glyphosate and glyphosate-tolerant genetically modified crops (esp. soy). In countries like Argentina or Brazil, for instance, a glyphosate ban would imply a direct attack on the cultivation of glyphosate-tolerant crops. In Sri Lanka, in contrast, no glyphosate-tolerant GM crops are authorized and the glyphosate-dependence of the country’s agricultural sector was arguably perceived to be lower, at least by policymakers. Whether true or not, it was frequently argued by politicians that large-scale organic agriculture would be feasible.

In the immediate aftermath of the glyphosate ban, its architects pushed for further policy advancement along the same lines. This was aided by the Sirisena government’s clear victory in the (early) parliamentary elections of August 2015, which renewed the government’s mandate (S45). In March 2016, the government unveiled the “Toxin-Free Nation” program, a 3-year plan aiming to “phase out” (all) pesticides and chemical fertilizers and to support a large-scale transition from “agrochemical” to “organic” agriculture (S46, S47, p. 16). The program was developed and managed by the Strategic Enterprise Management Agency, an agency that was directly linked to the Presidential Secretariat (S48) and lead by Asoka Abeygunawardana, a JHU activist and ally of Rathena Thero (S49). During the launch of the program, President Sirisena explicitly credited Rathana Thero as its “founder” (S50). Indeed, Rathana Thero continued to advocate for strict and comprehensive regulation, “seeking to extend the [glyphosate] ban to pesticides and chemical fertilisers” (S51). While the “Toxin-Free Nation” program was introduced *after* the 2015 glyphosate ban, the circumstances of its introduction clearly corroborate the central role that the policy preferences of Rathana Thero played in shaping the Sirisena government’s pesticide policy. Moreover, the “Toxin-Free Nation” program is arguably also the direct precursor of the more recent and much more consequential agrochemical ban introduced in 2021 by the subsequent Gotabaja Rajapaksa government.

In this section, we have used process tracing and a multiple-streams framework to explain the adoption of Sri Lanka’s 2015 glyphosate ban. We have demonstrated that glyphosate regulation entered the Sri Lankan political agenda as a potential solution to a very specific problem, namely the epidemic of CKDu. The problem stream of this epidemic and the policy stream of glyphosate regulation were coupled by the research of an activist health scientist. Despite high issue salience, an initial attempt at glyphosate regulation in 2014 failed due to limited electoral pressure and substantial business opposition, resulting in reduced political will. But a policy window for glyphosate regulation opened in 2015, after a political realignment brought to power a reformist government and with it, with elevated influence, a small Buddhist party with strong environmentalist policy preferences. The glyphosate ban of June 2015 resulted from the coupling of the policy and politics streams through the (parliamentary) leader of this Buddhist party, who essentially secured the new government’s commitment to (among other things) a glyphosate ban in exchange for his party’s political support. The adoption of Sri Lanka’s 2015 glyphosate ban can therefore be explained well with a multiple streams framework and particular reference to two distinct policy entrepreneurs.

### The glyphosate ban’s partial reversal in 2018

While Sri Lanka was the first country in the world to adopt and implement a full ban on glyphosate, the ban soon displayed a lack of “political sustainability” [57]. On the one hand, enforcement of the ban was insufficient, as evidenced by the continued use of previously imported and stocked glyphosate as well as the development of a significant black market (S52-54). On the other hand, the ban was partially reversed in May 2018, when a three-year permission to use glyphosate was granted to Sri Lanka’s tea and rubber sectors (S55-56). It is relevant to note that this temporary reversal was justified by the government to give the plantation sector more time to adapt, but it clearly also held the potential for a more permanent reversal. In this section we explain this partial reversal of Sri Lanka’s pioneering glyphosate ban by drawing on the concepts of self-undermining policy feedback [33] and problem-solution decoupling [77].

Sri Lanka’s glyphosate ban soon began to generate losses for the country’s agricultural sector, as the ban significantly increased the cost of weed control and thus overall production cost ([47], p. 249). These “emergent losses for organized groups” arguably set in motion “self-undermining feedback effects” ([33], pp. 445, 448). While small-scale rice farmers were likely also affected, it was mostly Sri Lanka’s well-organized and export-oriented plantation sector that engaged in publicizing these losses and advocating for a reversal of the ban. Sri Lanka’s Planters’ Association, the Ministry of Plantation Industries as well as the Tea Board were among the most vocal advocates for a reversal, regularly framing the glyphosate ban as a fundamental threat to the country’s large tea sector (S57-62). While much less visible than the plantation sector, the pesticide industry also supported these advocacy efforts. CropLife, an agrochemical industry group that also represents Monsanto/Bayer, funded a detailed empirical evaluation of the ban, which—unsurprisingly, given the clear conflict of interest—concluded that “banning glyphosate […] has pulled the food crop sector in to a catastrophe” (S63, pp. i, 35). CropLife also prepared a “one-pager” that concisely summarized their study’s main findings (S64).

A second factor that arguably also contributed to the postenactment contestation and eventual reversal of the ban, although in a less publicly discussed and therefore less visible manner, was the failure of the scientific community to convincingly corroborate the hypothesized link between glyphosate and CKDu, which had initially motivated Sri Lanka’s glyphosate ban in 2014. After the sudden coupling of CKDu and glyphosate in 2014 and the resulting glyphosate ban in 2015, the World Health Organization in Sri Lanka felt under pressure to justify its noncommittal, skeptical stance with regard to glyphosate’s relationship with CKDu [34].^8^ In April 2016, together with the Presidential Task Force on CKDu, the WHO therefore convened an international expert consultation on CKDu in Colombo (S65, p. 7; see [43]). Major conclusions of the consultation included calls for a uniform case definition of CKDu, better surveillance systems and more long-term research on risk factors (S65 p. 30). With regard to the hypothesized link between glyphosate and CKDu, the final report explicitly noted that the “evidence of causality of this association was considered inconclusive due to lack of consistency of the findings, lack of temporality where association was observed and limitations in the methods used for measuring exposures” (S65, p. 16). The WHO’s expert consultation therefore implicitly questioned the government’s own rationale for its glyphosate ban. President Sirisena must have been aware of these findings, given that his Presidential Task Force co-hosted the workshop and that its report was later presented to him personally (S66). While Channa Jayasumana, the policy entrepreneur who originally linked glyphosate with CKDu, still claimed in 2018 that “it has been proven beyond doubt, glyphosate is linked to chronic kidney disease epidemic in Sri Lanka” (S55), this view was not shared by CKDu experts or the WHO, thus somewhat “decoupling” ([77], p. 77) CKDu as a problem from glyphosate regulation as a solution.

A first focusing event that contributed toward the government’s decision to relax the glyphosate ban was the defeat of the governing coalition, and President Sirisena’s party in particular, in Sri Lanka’s local elections of February 2018. Nationwide, Sirisena’s party received only 12% of the vote, while the newly formed party of former president Mahinda Rajapaksa clearly won the election with 40%. Sirisena’s party even narrowly lost the district of Polonnaruwa, Sirisena’s own electoral district in the heartland of the CKDu epidemic. This crushing defeat put the government “in soul-searching mode, struggling to […] make changes in time to make sure Mr. Rajapaksa’s party does not ride discontent with the government to victory in the presidential election in 2019” (S67). Indeed, soon after the elections, in March 2018, President Sirisena appointed a special committee to study the “glyphosate issue” (S68) and to consider in particular plantation minister Navin Dissanayake’s proposal “to lift the ban on glyphosate for the plantation industry” (S69). While Rathana Thero and Sri Lanka’s health minister, Rajitha Senaratne, continued to strictly oppose any potential relaxation of the ban (S70), and were explicitly invited by Sirisena to be heard by the committee (S71), the establishment of this committee clearly signaled that President Sirisena was open to reconsider his strict stance on glyphosate.

The second focusing event that appears to have contributed toward the government’s decision to relax the glyphosate ban were extensive reports about potential Japanese restrictions on Sri Lankan tea imports, which helped to make economic concerns about the tea sector more salient. This issue, which was first reported in the Sri Lankan media in January 2018 (S72), was about the rejection of several shipments of Sri Lankan tea by Japanese authorities for exceeding the permitted Maximum Residue Levels of the herbicide MCPA, on which Sri Lankan tea planters had purportedly increasingly relied following the glyphosate ban. It is unclear to what extent the government’s glyphosate ban as such (rather than, for instance, poorly planned adaptation by tea planters) was actually responsible for this incidence and how much of an economic problem it really was, given that Japan, in early 2015, was only the ninth largest importer of Sri Lankan tea (S73). But such considerations were not taken into account in most media reports, with one suggesting that “the short sighted decision made by the authorities in banning Glyphosate weedicide in Sri Lanka will result in the loss of another major market for Ceylon Tea in Japan” (S74). The same narrative was supported by the plantation sector and the associated ministry.

Under the impression of the crushing election defeat as well as continued reports about the potential economic repercussions of the glyphosate ban, and in an apparent need to balance the demands of the pro- and anti-glyphosate factions within his government, President Sirisena eventually sought a “compromised approach” (S75). In May 2018, Sirisena’s cabinet decided to lift the glyphosate ban for Sri Lanka’s tea and rubber plantations (S76-77), which essentially followed the recommendation of plantation minister Dissanayake (S55, S78), but only under the relatively strict conditions developed by the above-mentioned special committee. The sectoral exception was to be introduced for “only” 36 months and its implementation was conditional on the development of a “monitoring mechanism” to “prevent misuse and leakage to water sources” (S75, S79). Details of this mechanism were clarified in September 2018 (S80) and controlled importation of glyphosate eventually began in December 2018 (S81).

The Sirisena government made clear, however, that it did not see this decision as a fundamental reversal of its glyphosate ban but rather as a—belatedly introduced—decision to implement it more gradually. According to health minister Rajitha Seneratne, the government still planned to “phase out the use of glyphosate by 2022, emulating similar measures taken by the European Union” (S82). At the same time, more state support for the development of an organic alternative to glyphosate was announced (S83). As of late 2018, where our systematic empirical analysis ends, glyphosate therefore remained banned outside of the tea and rubber sectors and, barring any further changes, Sri Lanka remained on track to (again) become the only country in the world with a complete glyphosate ban.

### Epilogue: crisis politics and the glyphosate ban’s full reversal in 2022

Sri Lanka’s broader political context has changed dramatically since 2018 [17, 18]. As a result, glyphosate regulation has become deeply enmeshed in broader crisis politics. Following a constitutional crisis in late 2018 and a series of Islamist terror bombings on Easter Sunday 2019, the Sirisena government was severely weakened ahead of upcoming presidential elections [17]. In November 2019, Gotabaya Rajapaksa was elected president, appointing his older brother Mahinda Rajapaksa—himself president from 2005 to 2015—as his prime minister. Ever since 2019, Sri Lanka has been in a deep economic crisis, driven, among other factors, by a series of November 2019 tax cuts and by the tourism-halting Covid-19 pandemic, both of which slashed government revenues (S84).

From the beginning of his presidency, Gotabaya Rajapaksa made clear that he supported strict agrochemical regulation. In his first address to parliament, in January 2020, he announced “plans to encourage Sri Lanka’s entire agriculture sector to shift to using only organic fertilizer within the next decade” (S85). On 27 April 2021, Rajapaksa’s cabinet passed a ban on the importation of *all* chemical fertilizers and pesticides. But rather than implementing it gradually, this blanket ban became effective almost immediately, on 6 May 2021 (S86-88).

Some observers have claimed that this radical policy shift toward organic agriculture was influenced by the Indian environmental activist Vandana Shiva ([53], S89). While we have not analyzed this policy episode in full detail, it appears that two other factors have contributed more significantly to motivating Sri Lanka’s 2021 agrochemical ban. First and most immediately, the Rajapaksa government apparently saw this large-scale import ban as an opportunity to alleviate foreign-exchange shortage (S90). Second, the Rajapaksa government was advised by the same policy entrepreneurs who had previously pushed for the 2015 glyphosate ban. In particular, Rathana Thero aligned himself with Gotabaya Rajapaksa during the latter's presidential campaign and vowed to “assist the President in achieving his vision” (S91-92). Channa Jayasumana was a member of the presidential task force that advised Rajapaksa on the implementation of the agrochemical ban and would later become a minister in his government (S93-94). This suggests that the 2021 agrochemical ban was driven by similar political forces as the 2015 glyphosate ban, albeit in a markedly different context.

Whatever its political causes were, the abrupt agrochemical ban of 2021 worsened the country’s ongoing economic crisis by impeding local food production and further driving up food prices [46]. The ban was eventually revoked after just 6 months, in November 2021, but the separate glyphosate ban remained in place (S95-96). The economic crisis continued to deepen and Sri Lanka defaulted on its sovereign debt in May 2022 ([18], p. 94). Severe food and fuel shortages triggered mass protests against the government until Gotabaya Rajapaksa was forced to resign and flee the country in July 2022. The government of his parliament-appointed successor, Ranil Wickremesinghe, has since passed a series of reforms focused on economic recovery and food security. These reforms have also included a full reversal of Sri Lanka’s 2015 glyphosate ban in August 2022 (S97-98). For the time being, this marks the end of Sri Lanka’s experiment with highly restrictive glyphosate regulation.

## Discussion

In discussing the broader implications of our findings for the politics of glyphosate regulation, we focus on the events between 2014 and 2018. The subsequent events, most notably Sri Lanka’s 2021 attempt to radically shift toward organic agriculture with a blanket ban on agrochemicals, are also important and our findings can contribute to a better understanding of the political origins of Sri Lanka’s recent crisis. But these post-2018 events can arguably tell us less about the political dynamics of glyphosate regulation per se, which is our primary theoretical interest.

Our analysis demonstrates that IARC’s 2015 classification of glyphosate as “probably carcinogenic to humans” did not play a crucial role in placing glyphosate on the Sri Lankan political agenda. This contrasts with European cases, where the IARC decision triggered “widespread concern” and motivated various political actors to reconsider their stance on glyphosate regulation ([67], p. 6; [66], p. 237). Agenda-setting in Sri Lanka was instead driven by more local dynamics, most notably critical research by Channa Jayasumana, an “activist scientist” [32] comparable to Gilles-Éric Séralini. Jayasumana’s work was strongly influenced by “local knowledge” [6], including principles of indigenous medicine. While this compromised the credibility of his claims in the “Western” science community, both in and outside Sri Lanka, it arguably amplified the claims’ local political impact (see [61]). This finding expands on previous research on the local development of glyphosate-critical “counter-expertise” in the Global South [1].

The adoption of Sri Lanka’s 2015 glyphosate ban clearly depended on the exceptionally high salience that glyphosate regulation acquired after it was coupled with the already highly salient epidemic of CKDu. Notably though, the health issue of CKDu and its coupling with agrochemical regulation is not unique to Sri Lanka. CKDu is also highly prevalent across Central America and was declared a “major public health problem” during a 2013 meeting of Central American health ministers (S99). In El Salvador, for instance, “kidney disease was the second most common cause of death among males in 2009” ([73], p. 1927), and local policy entrepreneurs linked this health problem with high agrochemical exposure in agricultural communities (S99). In response, the Salvadoran parliament passed a law in 2013 banning a series of agrochemicals, including (but not singularly focused on) glyphosate. However, this law was never signed by the president and thus never implemented (S99). Beyond Central America and South Asia, the main health risk usually invoked in connection with glyphosate, especially since the 2015 IARC decision, is non-Hodgkin lymphoma, a disease that is generally less prevalent and less politically salient than CKDu has been in countries like El Salvador and Sri Lanka.

The adoption of the Sri Lankan glyphosate ban also depended on the “de-delegation” [55] of glyphosate regulation. Until the early 2010s, Sri Lankan pesticide regulation was largely delegated to the Office of the Registrar of Pesticides, which has been described as operating “free from political interference” but in “constant dialogue with industry” ([59], p. 64). This system of “delegated policymaking” produced some significant policy achievements, in particular the 2008 decision to phase out the use of the highly toxic herbicide paraquat [59]. In 2014, however, the Registrar of Pesticides openly opposed the idea of a glyphosate ban, arguing that a link between glyphosate and CKDu, as suggested by Jayasumana, “is an interesting hypothesis, but we don’t have any evidence for it” (S16). When the glyphosate ban was eventually introduced in 2015, it was implemented through a ministry-level import ban that bypassed the Registrar of Pesticides. In other words, strict glyphosate regulation became possible after decision-making was *de*-delegated, moving from the bureaucratic to the cabinet level. This finding resonates with recent developments in the European Union, where pressure for stricter glyphosate regulation has come from the potentially more political Standing Committee on Plants, Animals, Food and Feed rather than the more technocratic European Food Safety Authority [3, 67].

A central feature of the Sri Lankan glyphosate ban was its apparent lack of “political sustainability” [57], as it was partially reversed in 2018 and fully revoked in 2022. Our analysis suggests that the ban’s partial reversal was the result of continued lobbying by Sri Lanka’s export-oriented plantation sector and increased political concerns about potential negative effects of the ban on the country’s large and structurally powerful tea sector. One particular source of the agricultural sector’s sustained postenactment opposition seems to have been the perceived lack of cost-effective alternatives to glyphosate [see 11, 27]. This confirms the hypothesis of political sustainability researchers that “resistance rather than adaptation by losers is more likely when their adaptation costs are high […] [and] perceived to be persistent over time” ([57], pp. 1121–1122). These findings resonate with developments in other countries, including El Salvador, France and Thailand, where strong political commitments to banning glyphosate were derailed before actual bans could be adopted or implemented (S99-101). Future research should therefore not only investigate if and why other countries can replicate the Sri Lankan policy experiment of banning glyphosate, but also if and why future glyphosate bans might prove more durable.

Based on journalistic reporting on the cases of Thailand and Mexico (S101-102), one might have expected pesticide industry lobbying to have played a central role in the reversal of the Sri Lankan glyphosate ban. Indeed, our analysis shows that the industry group CropLife funded a 2017 empirical evaluation that sought to demonstrate that “banning glyphosate […] has pulled the food crop sector in to a catastrophe” (S63, p. 35). Beyond this, however, we found no publicly available evidence of the involvement of the pesticide industry in the reversal of the glyphosate ban. Indeed, the fact that the partial reversal in 2018 benefited only the tea and rubber sectors strongly suggests that it was the influence of Sri Lanka’s export-oriented plantation sector, rather than the transnational pesticide industry, that was decisive in bringing about this reversal. However, this finding might also be a methodological artifact, as our analysis is limited to publicly availably documents. Future research could use freedom-of-information requests [42] to obtain documents from relevant regulatory agencies (e.g., the United States Trade Representative) that can provide insights on the transnational pesticide industry’s involvement in the Sri Lankan policy process. Such requests should be combined with in-depth interviews to assess the effect of such potential involvement on Sri Lankan government decisions [21].

## Conclusion

In this article, we have conducted a detailed case study of Sri Lanka’s pioneering but short-lived 2015 glyphosate ban. The Sri Lankan case provides a unique opportunity to examine both the political enablers of and barriers to strict glyphosate regulation. On the one hand, our analysis suggests that strict glyphosate regulation becomes more likely when it gets coupled with locally salient health risks and when decision-making authority is de-delegated from regulatory agencies back to the political executive. On the other hand, the short-lived nature of the Sri Lankan ban suggests that strict glyphosate regulation faces political sustainability threats, as the apparent lack of cost-effective alternative herbicides motivates persistent business lobbying for regulatory reversal.

In line with recent calls for more social science scholarship on the “global pesticide complex” [45] and the sociopolitical construction of “alternatives to glyphosate” [27], we see a great need for more research on the global politics of glyphosate regulation. For the countries for which failed glyphosate bans are already well documented, including El Salvador and Thailand, we need more systemic research on why these governments decided to give up on their planned bans. And for countries that continue to prepare plans for banning glyphosate, including Germany (S103) and Mexico (S104), it will be crucial to monitor concrete policy changes and explain why these countries might be able to follow through or else also give up on their planned bans.

### Supplementary Information

**Additional file 1.** Primary Sources.

## Data Availability

References to all primary data sources are included in the supplementary information file.

## Abbreviations

CKDu: Chronic Kidney Disease of Unknown Origin
EFSA: European Food Safety Authority
EPA: Environmental Protection Agency (United States)
EU: European Union
GDP: Gross Domestic Product
GM: Genetically Modified
GMO: Genetically Modified Organism
IARC: International Agency for Research on Cancer
JHU: National Heritage Party (*Jathika Hela Urumaya*)
MCPA: 2-Methyl-4-chlorophenoxyacetic acid
UN: United Nations
WHO: World Health Organization

## Footnotes

1 Technically, the chemical glyphosate is only the active ingredient in commercial glyphosate-based herbicide formulations, which also contain a variety of other, supposedly inert ingredients. While this fact is important in debates over the safety of glyphosate-based herbicides, we use the term glyphosate as a shorthand for glyphosate-based herbicide formulations.

2 Complete references to all our primary sources can be found in the Additional file 1.

3 The paper was subsequently republished in *Environmental Sciences Europe* [63].

4 While the republished Séralini paper was included in IARC’s review, the responsible working group eventually “concluded this was an inadequate study for the evaluation of glyphosate carcinogenicity” (S3, p. 357).

5 Jayasumana would later become a prominent national politician, winning a parliamentary seat in the North Central Province (2020) and becoming a minister in the government of Gotabaya Rajapaksa (2020–2022).

6 Jayasumana’s research on glyphosate would later also receive significant international attention. In 2016, Jayasumana testified at the civil society-organized (legally non-binding) International Monsanto Tribunal in The Hague (S19, p. 9), as did Séralini [8]. In 2019, Jayasumana, together with his PhD supervisor and co-author Sarath Gunatilake, received the Scientific Freedom and Responsibility Award from the American Association for the Advancement of Science (AAAS), a decision that was initially suspended, “after concerns were voiced by scientists and members” (S20), but eventually upheld (S21).

7 In December 2014, in the midst of this snap presidential campaign, the Mahinda Rajapaksa government actually introduced a regional ban on the sale and use of glyphosate (primarily) in the CKDu-affected North Central Province (S23). The ban clearly seemed motivated by short-term electoral calculations and it’s unlikely that it would have been enforced if Rajapaksa had won.

8 On the surface, the WHO’s skepticism in this regard may seem contradictory, as IARC, a WHO agency, had classified glyphosate as “probably carcinogenic to humans” in March 2015. The underlying issues, however, were distinct, as the IARC decision did not take into account potential links with CKDu (as it is not a cancer), while glyphosate’s potential carcinogenicity had not been a central issue in informing Sri Lanka’s glyphosate ban.

## References

[1] Arancibia F, Motta R (2019). Undone science and counter-expertise: fighting for justice in an Argentine community contaminated by pesticides. Sci Cult.

[2] Bacon MH, Vandelac L, Gagnon MA, Parent L (2023). Poisoning regulation, research, health, and the environment: the glyphosate-based herbicides case in Canada. Toxics.

[3] Bazzan G, Migliorati M (2020). Expertise, politics and public opinion at the crossroads of the European Commission’s decision-making: the case of Glyphosate. Int Rev Public Policy.

[4] Benbrook CM (2016). Trends in glyphosate herbicide use in the United States and globally. Environ Sci Eur.

[5] Benbrook CM (2019). How did the US EPA and IARC reach diametrically opposed conclusions on the genotoxicity of glyphosate-based herbicides?. Environ Sci Eur.

[6] Boossabong P (2017). Policy analysis in Thailand: comparing the roles of expert and local knowledge. J Comp Policy Anal Res Pract.

[7] Bozzini E (2020). Contrasting norms on the use of evidence in risk assessment: the controversy surrounding the carcinogenicity of glyphosate. Health Risk Soc.

[8] Busscher N, Colombo EL, van der Ploeg L, Gabella JI, Leguizamón A (2020). Civil society challenges the global food system: the International Monsanto Tribunal. Globalizations.

[9] Butler D (2012). Hyped GM maize study faces growing scrutiny. Nature.

[10] Chatterjee R. Mysterious kidney disease goes global. Science; 2016. https://www.science.org/content/article/mysterious-kidney-disease-goes-global.

[11] Clapp J (2021). Explaining growing glyphosate use: the political economy of herbicide-dependent agriculture. Glob Environ Chang.

[12] Cressey D (2015). Widely used herbicide linked to cancer. Nature.

[13] Culpepper PD (2011). Quiet politics and business power: corporate control in Europe and Japan.

[14] Dedieu F (2022). Organized denial at work: the difficult search for consistencies in French pesticide regulation. Regul Gov.

[15] Deegalle M (2004). Politics of the Jathika Hela Urumaya monks: Buddhism and ethnicity in contemporary Sri Lanka. Contemp Buddhism.

[16] Devapriya U. The crisis in Sri Lanka: economic and political dimensions. J Indo-Pac Aff. 2022. https://perma.cc/HZ9F-7Y5F.

[17] DeVotta N (2021). Sri Lanka: the return to ethnocracy. J Democr.

[18] DeVotta N (2022). Sri Lanka’s agony. J Democr.

[19] DeVotta N, Stone J (2008). Jathika Heia Urumaya and Ethno-Religious Politics in Sri Lanka. Pac Aff.

[20] Dewasiri N (2018). Jathika Chinthanaya: history and political significance. ColomboArts.

[21] Dorlach T, Mertenskötter P (2020). Interpreters of international economic law: corporations and bureaucrats in contest over Chile’s nutrition label. Law Soc Rev.

[22] Duke SO, Powles SB (2008). Glyphosate: a once-in-a-century herbicide. Pest Manag Sci.

[23] Gerring J, Cojocaru L (2016). Selecting cases for intensive analysis: a diversity of goals and methods. Sociol Methods Res.

[24] Gertler AL, Bullock JG (2017). Reference rot: an emerging threat to transparency in political science. PS Polit Sci Polit.

[25] Gillam C (2017). Whitewash: the story of a weed killer, cancer, and the corruption of science.

[26] Glenna L, Bruce A (2021). Suborning science for profit: Monsanto, glyphosate, and private science research misconduct. Res Policy.

[27] Goulet F, Aulagnier A, Fouilleux E (2023). Moving beyond pesticides: exploring alternatives for a changing food system. Environ Sci Policy.

[28] Gunnell D, Fernando R, Hewagama M, Priyangika WDD, Konradsen F, Eddleston M (2007). The impact of pesticide regulations on suicide in Sri Lanka. Int J Epidemiol.

[29] Guyton KZ, Loomis D, Grosse Y, El Ghissassi F, Benbrahim-Tallaa L, Guha N (2015). Carcinogenicity of tetrachlorvinphos, parathion, malathion, diazinon, and glyphosate. Lancet Oncol.

[30] Hendlin YH, Arcuri A, Lepenies R, Hüesker F (2020). Like oil and water: the politics of (not) assessing glyphosate concentrations in aquatic ecosystems. Eur J Risk Regul.

[31] Howlett M (2022). Looking at the ‘field’ through a Zoom lens: methodological reflections on conducting online research during a global pandemic. Qual Res.

[32] Isopp B. The perils, politics, and promises of activist science. In: Bencze L, Alsop S, editors. Activist science and technology education. Springer; 2014. p. 307–21.

[33] Jacobs AM, Weaver RK (2015). When policies undo themselves: self-undermining feedback as a source of policy change. Governance.

[34] Jayatilake N, Mendis S, Maheepala P, Mehta FR (2013). Chronic kidney disease of uncertain aetiology: prevalence and causative factors in a developing country. BMC Nephrol.

[35] Jayasumana C, Paranagama PA, Amarasinghe MD, Wijewardane KMRC, Dahanayake KS, Fonseka SI (2013). Possible link of chronic arsenic toxicity with chronic kidney disease of unknown etiology in Sri Lanka. J Nat Sci Res.

[36] Jayasumana C, Gunatilake S, Senanayake P (2014). Glyphosate, hard water and nephrotoxic metals: are they the culprits behind the epidemic of chronic kidney disease of unknown etiology in Sri Lanka?. Int J Environ Res Public Health.

[37] Jayasumana C, Paranagama P, Agampodi S, Wijewardane C, Gunatilake S, Siribaddana S (2015). Drinking well water and occupational exposure to herbicides is associated with chronic kidney disease, in Padavi-Sripura, Sri Lanka. Environ Health.

[38] Kay A, Baker P (2015). What can causal process tracing offer to policy studies? A review of the literature. Policy Stud J.

[39] Kingdon JW (1984). Agendas, alternatives, and public policies.

[40] Kinniburgh F (2023). The politics of expertise in assessing alternatives to glyphosate in France. Environ Sci Policy.

[41] Knipe DW, Chang SS, Dawson A, Eddleston M, Konradsen F, Metcalfe C, Gunnell D (2017). Suicide prevention through means restriction: impact of the 2008–2011 pesticide restrictions on suicide in Sri Lanka. PLoS ONE.

[42] Krimsky S, Gillam C (2018). Roundup litigation discovery documents: implications for public health and journal ethics. J Public Health Policy.

[43] Kumaresan J, Seneviratne R (2017). Beginning of a journey: unraveling the mystery of chronic kidney disease of unknown aetiology (CKDu) in Sri Lanka. Glob Health.

[44] Lim TC (2016). Doing comparative politics: an introduction to approaches and issues.

[45] Mansfield B, Werner M, Berndt C, Shattuck A, Galt R, Williams B (2023). A new critical social science research agenda on pesticides. Agric Hum Values.

[46] Marambe B (2022). Sri Lanka needs a return to “good agricultural practices”. Organ Prof Assoc Sri Lanka.

[47] Marambe B, Herath S (2020). Banning of herbicides and the impact on agriculture: the case of glyphosate in Sri Lanka. Weed Sci.

[48] McHenry LB (2018). The Monsanto Papers: poisoning the scientific well. Int J Risk Saf Med.

[49] Motta R, Arancibia F, Chamberlain JM (2015). Health experts challenge the safety of pesticides in Argentina and Brazil. Medicine, risk, discourse and power.

[50] Moravcsik A (2014). Transparency: the revolution in qualitative research. PS Polit Sci Polit.

[51] Myers JP, Antoniou MN, Blumberg B, Carroll L, Colborn T, Everett LG (2016). Concerns over use of glyphosate-based herbicides and risks associated with exposures: a consensus statement. Environ Health.

[52] Nakandala D, Turpin T. The barriers to innovation diffusion: the case of GM food in Sri Lanka. In: Urban agriculture and food systems: breakthroughs in research and practice. Hershey: IGI Global; 2019. p. 49–66.

[53] Nordhaus T, Shah S. In Sri Lanka, organic farming went catastrophically wrong. Foreign Policy; 2022. https://perma.cc/GLT6-4XC5.

[54] Ollinaho OI, Pedlowski MA, Kröger M (2023). Toxic turn in Brazilian agriculture? The political economy of pesticide legalisation in post-2016 Brazil. Third World Q.

[55] Ozel I (2012). The politics of de-delegation: regulatory (in) dependence in Turkey. Regul Gov.

[56] Paskalev V (2020). The clash of scientific assessors: what the conflict over glyphosate carcinogenicity tells us about the relationship between law and science. Eur J Risk Regul.

[57] Patashnik EM, Weaver RK (2021). Policy analysis and political sustainability. Policy Stud J.

[58] Pearce N, Blair A, Vineis P, Ahrens W, Andersen A, Anto JM (2015). IARC monographs: 40 years of evaluating carcinogenic hazards to humans. Environ Health Perspect.

[59] Pearson M, Zwi AB, Buckley NA, Manuweera G, Fernando R, Dawson AH, McDuie-Ra D (2015). Policymaking ‘under the radar’: a case study of pesticide regulation to prevent intentional poisoning in Sri Lanka. Health Policy Plan.

[60] Pett J, Mohamed F, Knight J, Linhart C, Osborne NJ, Taylor R (2022). Two decades of chronic kidney disease of unknown aetiology (CKDu) research: existing evidence and persistent gaps from epidemiological studies in Sri Lanka. Nephrology.

[61] Rambukwella H (2023). Patriotic science: the COVID-19 pandemic and the politics of indigeneity and decoloniality in Sri Lanka. Interventions.

[62] Senanayake N (2022). “We spray so we can live”: agrochemical kinship, mystery kidney disease, and struggles for health in dry zone Sri Lanka. Ann Am Assoc Geogr.

[63] Séralini GE, Clair E, Mesnage R, Gress S, Defarge N, Malatesta M (2014). Republished study: long-term toxicity of a Roundup herbicide and a Roundup-tolerantgenetically modified maize. Environ Sci Eur.

[64] Shattuck A (2021). Generic, growing, green?: the changing political economy of the global pesticide complex. J Peasant Stud.

[65] Starke P (2013). Qualitative methods for the study of policy diffusion: challenges and available solutions. Policy Stud J.

[66] Tosun J, Debus M (2021). Right-wing populist parties and environmental politics: insights from the Austrian Freedom Party’s support for the glyphosate ban. Environ Polit.

[67] Tosun J, Lelieveldt H, Wing TS (2019). A case of ‘muddling through’? The politics of renewing glyphosate authorization in the European Union. Sustainability.

[68] Tosun J, Varone F (2021). Politicizing the use of glyphosate in Europe: comparing policy issue linkage across advocacy organizations and countries. J Comp Policy Anal Res Pract.

[69] Trampusch C, Palier B (2016). Between X and Y: how process tracing contributes to opening the black box of causality. New Political Econ.

[70] Van Bruggen AH, He MM, Shin K, Mai V, Jeong KC, Finckh MR, Morris JG (2018). Environmental and health effects of the herbicide glyphosate. Sci Total Environ.

[71] Villnow V, Rombach M, Bitsch V (2019). Examining German media coverage of the re-evaluation of glyphosate. Sustainability.

[72] Werner M, Berndt C, Mansfield B (2022). The glyphosate assemblage: herbicides, uneven development, and chemical geographies of ubiquity. Ann Am Assoc Geogr.

[73] Wesseling C, Crowe J, Hogstedt C, Jakobsson K, Lucas R, Wegman DH (2013). The epidemic of chronic kidney disease of unknown etiology in Mesoamerica: a call for interdisciplinary research and action. Am J Public Health.

[74] Widger T (2021). Glyphosate regulation and sovereignty politics around the world. Anthropol Today.

[75] Yin RK (2018). Case study research and applications.

[76] Zahariadis N. The multiple streams framework: structure, limitations, prospects. In: Sabatier PA, editor. Theories of the policy process. Westview Press; 2007. p. 65–92.

[77] Zahariadis N, Exadaktylos T (2016). Policies that succeed and programs that fail: ambiguity, conflict, and crisis in Greek higher education. Policy Stud J.

