# Supplementary material for: The politics of glyphosate regulation: lessons from Sri Lanka’s short-lived ban
Source: Global Health. 2023 Nov 13;19:84. doi: 10.1186/s12992-023-00981-2 (PMC10644602; doi:10.1186/s12992-023-00981-2)
Supplement: Supplementary file 1 — Additional file 1. Primary Sources. [file 12992_2023_981_MOESM1_ESM.pdf]

Additional file 1

The politics of glyphosate regulation: lessons from Sri Lanka's short-lived ban

Tim Dorlach & Sandya Gunasekara

Globalization and Health

DOI: https://doi.org/10.1186/s12992-023-00981-2

Primary Sources

Source 1: Cohen, P. (2020, June 24). Roundup maker to pay $10 billion to settle cancer suits. The New York Times. Available at: https://perma.cc/XMY4-CHWE

Source 2: Pollack, A. (2012, September 19). Foes of modified corn find support in a study. The New York Times. Available at: https://perma.cc/8AKL-F84Q

Source 3: International Agency for Research on Cancer (2016). Some organophosphate insecticides and herbicides. IARC monographs on the evaluation of carcinogenic risks to humans, 112. Available at: https://perma.cc/9E3F-7ASK

Source 4: Foucart, S. & Horel, S. (2017, June 2). Monsanto papers. Le Monde, France. Available at: https://perma.cc/U7G6-SNB6

Source 5: Industry edited EFSA's Glyphosate evaluation ahead of publication. (2017, July 12). Corporate Europe Observatory. Available at: https://perma.cc/S86S-XS5K

Source 6: Neslen, A. (2017, September 14). EU report on weedkiller safety copied text from Monsanto study. The Guardian. Available at: https://perma.cc/7HA8-N9R8

Source 7: Brzezinski, B. (2023, July 17). Brussels says glyphosate safe enough for 'full' re-approval. Politico. Available at: https://perma.cc/NN8U-NMHX

Source 8: Marks, S. & Paravicini, G. (2017, November 29). French and Italians sense golden opportunity in glyphosate ban. Politico. Available at: https://perma.cc/LVK6-K22N

Source 9: Weir, D. (2015, May 15). Aerial use of glyphosate herbicides in Colombia prove too controversial after WHO findings on cancer risks. Conflict and Environment Observatory. Available at: https://perma.cc/53GN-2BK7

Source 10: Campaign to ban three hazardous chemicals in Thailand. (2018, September 9). BIOTHAI. Available at: https://perma.cc/ZNU3-2PL9

Source 11: Thailand reverses ban of chemicals use in pesticides. (2019, November 27). Reuters. Available at: https://perma.cc/UY7S-RL96

Source 12: Gillam, C. (2020, September 17). Thailand's reversal on glyphosate ban came after Bayer scripted U.S. intervention, documents show. U.S. Right to Know. Available at: https://perma.cc/TF76-LX66

Source 13: Department of Commerce (2015). International trade statistics of Sri Lanka – 2015. Available at: https://perma.cc/R5FJ-4DKN

Source 14: Gunawardene, N. (2014, August 15). When worlds collide #117: Once and future organics in Sri Lanka. WordPress. Available at: https://perma.cc/RC3T-NH4J

Source 15: Galappattige, A. (2018, November 29). Agricultural biotechnology report 2018. Global Agricultural Information Network. Available at: https://perma.cc/BQ7Y-7VHU

Source 16: Chavkin, S. (2014, April 11). Sri Lanka delays herbicide ban as kidney disease origin remains elusive. International Consortium of Investigative Journalists. Available at: https://perma.cc/SG7V-HT79

Source 17: MoD staff educated on the values of herbal drink. (2015, August 14). News.lk. Available at: https://perma.cc/FTG7-SFBY

Source 18: R. D. S. S. Wickramasinghe., K. S. Dahanayaka., M. P. Batagoda., G. V. C. P. de Silva., P. Senanayake., J. S. A. Pathirana., S. Sonnadara., & S. P. Saman Kumara (2019). Beneficial effects of Non-chemically Grown Traditional Rice/Rice Based Functional Food (NGTRF) in nutritional management of Nafld and diabetes mellitus. *Journal of Food Science and Engineering* 9, 15-44. Available at: https://perma.cc/64VL-QKZ4

Source 19: Jayasumana, C. (2016, August 16). Tribunal Monsanto. Available at: https://perma.cc/6JTU-5HVY

Source 20: Handunnetti, D. (2019, February 28). Sri Lanka scientist blames industry as award for herbicide research is axed. Mongabay. Available at: https://perma.cc/P3HA-4UZM

Source 21: Handunnetti, D. (2019, November 16). 'Science prevails' as suspension of award for herbicide research is reversed. Mongabay. Available at: https://perma.cc/N8QF-WTWN

Source 22: Chavkin, S. (2014, March 13). Sri Lanka bans leading Monsanto herbicide citing deadly disease fears. International Consortium of Investigative Journalists. Available at: https://perma.cc/UH6R-MX4W

Source 23: Sri Lanka bans pesticides containing 5 chemicals. (2014, December 23). Adaderana. Available at: https://perma.cc/E66J-5XXL

Source 24: Devapriya, U. (2015, January 13). A tribute to Athuraliye Rathana Thero. Colombo Telegraph. Available at: https://perma.cc/N4C5-SZG2

Source 25: Robin, M. (2018, August 18). Menace sur le thé sri lankais bientôt imbibé de glyphosate. Le blog de Marie-Monique Robin. Available at: https://perma.cc/U7ZP-CT8W

Source 26: Government announces allowance for Sri Lanka's kidney patients after opposition candidate sets up fund. (2014, December 09). Sri Lanka Financial Chronicle. Available at: https://perma.cc/VMW8-CCKM

Source 27: Sirisena, M. (2014). A compassionate Maithri, governance, a stable country. New Democratic Front. Groundviews. Available at: https://perma.cc/MQP7-258A

Source 28: Maithripala Sirisena's 100 day work programme; Detailed diary description. (2014, December 20). Colombo Telegraph. Available at: https://perma.cc/H8ZU-TNTM

Source 29: Sirilal, R. & Busvine, D. (2015, August 16). Sri Lankans to elect parliament in 'referendum' on Rajapaksa comeback. Reuters. Available at: https://perma.cc/4DPG-WDKB

Source 30: President distributes Rs.3000 assistance to kidney patients. (2015, January 26). News.lk. Available at: https://perma.cc/HE2V-ANMK

Source 31: Business community support National Kidney Trust Fund. (2015, February 26). News.lk. Available at: https://perma.cc/FNB8-KYZ8

Source 32: President to open 'Methsiri Sevana', the Kidney Patients Relief Centre today. (2019, March 20). News.lk. Available at: https://perma.cc/LW8L-DJHV

Source 33: Silva, N. (2020, February 5). UNP accuses govt of slashing CKD allowance. Economynext. Available at: https://perma.cc/K3N8-AXKQ

Source 34: President receives foreign support to control the kidney disease. (2015, July 30). News.lk. Available at: https://perma.cc/VVV4-CV3A

Source 35: China to help Sri Lanka fight chronic kidney disease. (2015, July 31). Gov.cn. Available at: https://perma.cc/EX4A-5HJQ

Source 36: Sri Lankan president opens China-funded nephrology hospital. (2021, June 12). Xinhuanet. Available at: https://perma.cc/8TX6-4F6T

Source 37: President says joint task force will be created to prevent kidney diseases. (2015, February 26). News 1st. Available at: https://perma.cc/ZCZ5-BY5W

Source 38: Hettiarachchi, K. (2014, April 27). Tackling kidney disease in NCP. The Sunday Times. Available at: https://perma.cc/T27Q-44ED

Source 39: Decisions taken at the cabinet meeting held on 27th May 2015. (2015, May 28). News.lk. Available at: https://perma.cc/H2RY-YVH8

Source 40: Agriculture department cancels permits to import glyphosate. (2015, June 1). News.lk. Available at: https://perma.cc/8WKJ-QNF3

Source 41: Govt. issues gazette notification banning glyphosate. (2015, June 15). News.lk. Available at: https://perma.cc/XP6V-G9VT

Source 42: Rodrigo, M. (2015, January 18). Environmentalists hope Sirisena will honour pledge. The Sunday Times. Available at: https://perma.cc/2T2M-55DG

Source 43: Glyphosate: Don't allow TNCs to run the government. (2016, September 23). Daily Mirror. Available at: https://perma.cc/RW3J-6SL7

Source 44: Devapriya, U. (2015, January 13). A tribute to Athuraliye Rathana Thero. Colombo Telegraph. Available at: https://perma.cc/N4C5-SZG2

Source 45: Barstow, D. (2015, August 18). Sri Lankans reject ex-president Mahinda Rajapaksa in election, and prosecution may follow. The New York Times. Available at: https://perma.cc/5YTM-KWT4

Source 46: Abeygunawardana, A. (2016, October 22). Wither the toxin-free nation. Colombo Telegraph. Available at: https://perma.cc/T498-UYWU

Source 47: Waidyanatha, P. (2021). 'Toxin-free nation' agriculture policy: Rhetoric & repercussions. Available at: https://perma.cc/2W2D-DMQ5

Source 48: The ill-fated road towards a toxin-free nation: Why good intentions alone are not enough for Sri Lanka's future food security. (2018, January 2). Echelon. Available at: https://perma.cc/78V2-6VUH

Source 49: Abeygunawardana, A. (2015). The revolution of the era: The inside story of how Maithri defeated Mahinda. Colombo. Available at: https://perma.cc/5SDN-QK7H

Source 50: Sirisena, M. (2016, March 8). "Vasa Visa Nethi Ratak" national program - President's speech (video file). Youtube. Available at: https://www.youtube.com/watch?v=Q9xdMXKvMis

Source 51: Jaysundera, D. (2016, August 15). Rathana Thero insists on blanket ban for all toxin treatments in agriculture. Daily Ft. Available at: https://perma.cc/APQ6-ZCQ2

Source 52: Sri Lanka customs seizes a container of banned glyphosate. (2017, September 25). AgNews. Available at: https://perma.cc/TCZ9-Y9W3

Source 53: Anuradhapura, N. (2016, September 8). Pesticides containing glyphosate being sold despite ban. Daily News. Available at: https://perma.cc/W93M-33WV

Source 54: Dhanapala, B. (2018, April 6). Ban the black market and permit the popular pesticide. Daily News. Available at: https://perma.cc/PD98-FDCJ

Source 55: Ariff, Y. (2018, May 2). Glyphosate ban lifted for tea and rubber. Adaderana. Available at: https://perma.cc/H9NM-H54N

Source 56: Glyphosate ban lifted finally. (2018, July 16). Daily Ft. Available at: https://perma.cc/WXU3-HMQA

Source 57: Fernandopulle, L. (2017, December 10). Ban on glyphosate: Planters' plea for an alternative. Sunday Observer. Available at: https://perma.cc/W796-3FVX

Source 58: Sri Lanka's glyphosate ban cost Rs35bn in crop losses: Farm managers. (2018, May 2). Economynext. Available at: https://perma.cc/NQ9Y-4LMK

Source 59: Allington, A. (2018, January 9). Sri Lankan tea farmers sense trouble brewing over glyphosate ban. Bloomberg Law. Available at: https://perma.cc/45HQ-JUTU

Source 60: Dias, S. (2017, September 24). Tea industry in limbo after glyphosate ban. The Sunday Times. Available at: https://perma.cc/9GY3-EAH7

Source 61: Plantation minister emphasizes the need for weedicides to prevent damage to tea industry. (2018, March 18). Colombo Page. Available at: https://perma.cc/6LBL-LH74

Source 62: Lifting of ban on glyphosate will rescue Lanka's tea and rubber plantations. (2018, February 23). News In Asia. Available at: https://perma.cc/7CDF-JJY5

Source 63: Abeywickrama, l., Sandika, A., Sooriyarachchi, P. & Vidanapathirana, I. (2017). Impacts of banning glyphosate on agriculture sector in Sri Lanka; A field evaluation. University of Ruhuna. Available at: https://perma.cc/SKP5-FEQG

Source 64: Impacts of the 2015 ban on glyphosate in Sri Lanka: Key findings from Abeywickrama (2018) field study. (2018). Crop Life Asia. Available at: https://perma.cc/7WKE-9U67

Source 65: International expert consultation on chronic kidney disease of unknown etiology (2016). World Health Organization, Country Office for Sri Lanka. Available at: https://perma.cc/S6RU-PBU2

Source 66: WHO expert consultation recommends measures to address chronic kidney disease. (2016, May 7). News.lk. Available at: https://perma.cc/R3Q7-XUET

Source 67: Mashal, M. & Bastians, D. (2018, February 11). Election losses test Sri Lanka's leader, and the country's direction. The New York Times. Available at: https://perma.cc/6QMH-H44H

Source 68: Cabinet commitee of experts to study glyphosate issue. (2018, March 28). News.lk. Available at: https://perma.cc/VQ4T-BDAN

Source 69: Ariff, Y. (2018, May 27). Cabinet to appoint expert committee on glyphosate. Adaderana. Available at: https://perma.cc/26ER-5RPW

Source 70: Sri Lankan government split on whether to lift ban on glyphosate herbicide. (2018, April 5). AgNews. Available at: https://perma.cc/MC7X-F58H

Source 71: Cabinet Commitee of experts to study glyphosate issue. (2018, March 28). News.lk. Available at: Available at: https://perma.cc/VQ4T-BDAN

Source 72: Ban on glyphosate and use of MCPA weedicide affects the tea industry in Sri Lanka. (2018, January 24). Daily Ft. Available at: https://perma.cc/P3XF-6XWR

Source 73: Sri Lanka March tea exports dip. (2015, April 24). Daily Mirror. Available at: https://perma.cc/QVR5-PM8J

Source 74: Subasinghe, C. (2018, January 30). Ban on glyphosate, use of MCPA weedicide affects tea industry. Daily News. Available at: https://perma.cc/DX9F-SMNE

Source 75: Senanayake, R. (2018, May 9). The glyphosate challenge. Colombo Telegraph. Available at: https://perma.cc/X262-TUSM

Source 76: Glyphosate ban lifted for tea & rubber only for 36 months. (2018, May 11). Daily Ft. Available at: https://perma.cc/7UFH-MAVW

Source 77: Cabinet nod to lift ban on glyphosate for 36 months. (2018, May 10). Daily Mirror. Available at: https://perma.cc/MQ4F-6XC9

Source 78: Govt to lift Glyphosate ban on tea, rubber industries. (2018, May 3). Daily News. Available at: https://perma.cc/KNP8-36U4

Source 79: Ariff, Y. (2018, May 10). Cabinet approval to lift ban on glyphosate for tea and rubber. Adaderana. Available at: https://perma.cc/YZC8-SC24

Source 80: Christopher, C. (2018, September 16). Plantations to get glyphosate soon. The Sunday Times. Available at: https://perma.cc/558D-8GKG

Source 81: Dias, S. (2018, December 23). Glyphosate back: Plantations would take 10 yrs to recover. The Sunday Times. Available at: https://perma.cc/5SCF-9L7D

Source 82: Glyphosate ban lifted for tea & rubber only for 36 months. (2018, May 11). Daily Ft. Available at: https://perma.cc/7UFH-MAVW

Source 83: Environment friendly substitute for glyphosate. (2018, July 17). News.lk. Available at: https://perma.cc/74MS-NPZR

Source 84: Adrogué, B.C. & Plant, M. (2023, February 8). Navigating troubled waters: Sri Lanka's default and the challenges ahead. Center for Global Development. Available at: https://perma.cc/TW4V-XBFG

Source 85: The policy statement made by H.E. (2020, January 3). Presidential Secretariat. Available at: https://perma.cc/8TT5-JL3F

Source 86: Importation of chemical fertilizers will be stopped completely …. (2021, April 22). Presidential Secretariat. Available at: https://perma.cc/3H3Y-V5LW

Source 87: President emphasizes need to eliminate use of chemical fertilizers. (2021, April 29). Adaderana. Available at: https://perma.cc/2WK3-AUTA

Source 88: Sri Lanka restricts and bans the import of fertilizers and agrochemicals. (2021, May 28). United States Department of Agriculture - Foreign Agricultural Service & Global Agricultural Information Network. Available at: https://perma.cc/78FN-2BQJ

Source 89: Verma, M. (2022, April 11). How environmental wokeness cost Sri Lanka its food security. News 18. Available at: https://perma.cc/9X9Z-K6F4

Source 90: Sri Lanka to ban import, use of fertilizer, agro-chemicals to save foreign exchange. (2021, April 30). Economynext. Available at: https://perma.cc/35WX-Z9KD

Source 91: Rathana Thera pledges support for Gotabaya. (2019, October 5). Adaderana. Available at: https://perma.cc/Q9DG-8D35

Source 92: Rathana Thero to work for president's vision. (2020, December 20). News 1st. Available at: https://perma.cc/Q2NQ-TLWK

Source 93: Presidential task force for a green socio-economy. (2021, May 10). Presidential Secretariat. Available at: https://perma.cc/GFF7-TN4S

Source 94: Sri Lanka drug manufacturers welcome pharma state ministry. (2020, August 13). Economynext. Available at: https://perma.cc/3H3A-SJFT

Source 95: Jayasinghe, U. (2021, November 24). Sri Lanka rows back on chemical fertiliser ban, but yields may not rebound. Reuters. Available at: https://perma.cc/2D6Q-KVF8

Source 96: Sri Lanka agriculture minister removes official who lifted glyphosate ban. (2021, November 23). Economynext. Available at: https://perma.cc/KKY8-PZCM

Source 97: Sri Lanka president lifts import ban on glyphosate. (2022, August 8). Economynext. Available at: https://perma.cc/25WT-T7FF

Source 98: Sri Lanka lifts import ban on glyphosate. (2022, August 13). Adaderana. Available at: https://perma.cc/H9S4-SRXM

Source 99: Reveley, F. (2022, November 16). In El Salvador and beyond, an unsolved kidney disease mystery. Undark. Available at: https://perma.cc/5L95-M9WJ

Source 100: Allemandou, S. (2018, September 23). Glyphosate, the issue poisoning Macron's government. France 24. Available at: https://perma.cc/RUN5-NHTX

Source 101: Gillam, C. (2020, September 17). Thailand's reversal on glyphosate ban came after Bayer scripted U.S. intervention, documents show. U.S. Right to Know. Available at: https://perma.cc/TF76-LX66

Source 102: Gillam, C. (2021, February 16). Revealed: Monsanto owner and US officials pressured Mexico to drop glyphosate ban. The Guardian. Available at: https://perma.cc/8Q6L-26D2

Source 103: Dahm, J. (2023, May 4). Berlin's promised glyphosate ban plans could face hurdles at EU level. Euractiv. Available at: https://perma.cc/E8K4-YUWV

Source 104: Erickson, B.E. (2023, February 16). Mexico revises plan to phase out glyphosate, biotech corn. C&en. Available at: https://perma.cc/33VM-BEE9
